# Breast-cancer specific comprehensive archive of Patient-Reported Outcome Measures (PROMs) for clinical research and clinical practice in oncology: Results from the PRO4All project

**DOI:** 10.1016/j.breast.2024.103817

**Published:** 2024-09-28

**Authors:** Anna Amela Valsecchi, Filippo Giovanardi, Francesco Malandrini, Michela Meregaglia, Alberto Servetto, Chiara Bennati, Carmine Pinto, Massimo Di Maio, Oriana Ciani

**Affiliations:** a Department of Oncology, University of Turin, A.O.U. Città della Salute e della Scienza di Torino, Ospedale Molinette, Turin, Italy; b Medical Oncology, Azienda Unità Sanitaria Locale-IRCCS, Reggio Emilia, Italy; c SDA Bocconi School of Management, Center for Research on Health and Social Care Management, Milan, Italy; d Department of Clinical Medicine and Surgery, University of Naples Federico II, Naples, Italy; e Oncology Unit, Ausl Romagna Ravenna, Emilia Romagna, Italy

**Keywords:** Patient-reported outcome measure, Health-related quality of life, Breast cancer, Outcome domains, Archive, PRO4All

## Abstract

**Background:**

Inclusion of patient-reported outcomes (PROs) in oncology clinical trials is strongly recommended. However, selecting the most appropriate patient-reported outcome measures (PROMs) is not easy. This study aimed to develop a breast cancer (BC) specific comprehensive archive of PROMs.

**Methods:**

As part of the PRO4All project, we identified available PROMs in oncology by searching facit.org, eortc.org, eprovide.mapi-trust.org, PubMed, ema.europa.eu (European Public Assessment Reports) and published reviews. For this analysis, only BC tools were extracted. We described information about PROM name, type of questionnaire, questionnaire variant(s), recall period, number of items, and presence of minimum clinically important difference (MCID) reference in literature. Then, we assigned each item to a specific domain according to a predefined taxonomy of 38 items for outcome classification.

**Results:**

We identified and analyzed 383 PROMs. Of these, 29 were BC specific, but 2 were excluded because the questionnaires description was not available. 6 (22.2 %) were variants of another questionnaire. All questionnaires were self-reported. In 6 cases (22.2 %) the recall period to consider was the “last week”. The mean number of items per questionnaire was 25.81 (range 6–71). 602 items were assigned to an outcome domain: emotional functioning/wellbeing in 26.6 % of cases, physical functioning in 14.1 %, delivery of care in 10.8 %, and general outcomes in 10.5 %. MCID reference was found only in 4 (14.8 %) cases.

**Conclusions:**

The newly developed archive represents a useful tool to optimize the use of PROMs in the evaluation of treatments in BC patients, promoting a patient-centered approach both in clinical research and practice.

## Highlights

• Inclusion of PROs in oncology clinical trials is strongly recommended.
• Selecting the most appropriate PROMs is not easy.
• This study developed a breast cancer specific comprehensive archive of PROMs.
• This tool promotes a patient-centered approach in clinical research and practice.

## 1 Introduction

Patient-reported outcomes (PROs) refer to the results or outcomes reported directly by the patients themselves [1], without being interpreted by others [2].

Patient-reported outcome measures (PROMs), developed to measure PROs, are essential tools in healthcare that involve questionnaires completed by patients, with the aim of assessing various aspects of their health without the influence or the filter by healthcare professionals [3,4]. These measures are crucial in capturing patients’ perspectives regarding their symptom burden, treatment impact, and health-related quality of life (QoL), making them valuable in clinical decision-making, patient-centered care, and health policy development.

Over the past three decades, there has been a growing focus on standardizing PROMs as performance measures, which are crucial for evaluating various health domains and assessing patient feasibility and acceptability [5]. PROMs are essential tools for capturing self-reported feelings and functions, including QoL (both global status and specific domains and scales), symptoms associated to disease and/or treatment toxicity [6].

As for their potential role in clinical practice, although PROMs have gained acceptability among patients and potential benefits in symptom detection [7] and clinician– patient communication, there is still a lack of definitive evidence on how clinicians can effectively utilize PROMs data to enhance service quality and improve patient-reported outcomes. Further research is needed to understand how to best incorporate these measures into routine clinical practice and address perceived barriers by health professionals [8,9].

Standardizing their use as performance measures enhances the evaluation of healthcare quality and patient outcomes across various medical specialties. Efforts to integrate PROMs into routine clinical practice and address implementation barriers are crucial for maximizing their potential benefits in improving patient care and outcomes [10].

Focusing on clinical research, assessing PROs is crucial for understanding the benefits, tolerability and impact on QoL of novel oncology drugs [11,12]. This is true both in industry-sponsored clinical trials, aimed at regulatory approval, and in academic studies, designed to optimize treatment choices in clinical practice.

PROs encompass several health-related assessments directly reported by patients. These outcomes can be categorized into different domains, including QoL [13,14] or health-related QoL [15], functional status, symptoms or symptom burden, health behaviors, and patient experience [16]. PROs are crucial in clinical trials because they provide insights into the physical, functional, and psychological consequences of treatment and the impact of disease symptoms [[17], [18], [19]].

These outcomes are collected [20] through standardized questions administered in various ways, including face-to-face interviews, telephone surveys, paper questionnaires, and digital platforms [21,22]. PROs can serve as endpoints in randomized controlled trials, emphasizing their importance in evaluating treatment effectiveness from the patient's perspective.

In recent years, there is growing interest in integrating electronic PROs (ePROs) both into routine oncology practice for symptom monitoring [23,24] and in clinical trials. This integration, especially in patients with breast cancer (BC), allows for a more patient-centered approach to care, thereby offering valuable insights into patient symptoms, treatment outcomes, and overall well-being. Integrating PROs into oncology clinical trials is highly advocated because of their potential to provide valuable insights into the patient's perspective from various aspects. However, selecting the most suitable PROMs for these trials presents significant challenges.

As part of PRO4AII project, we performed a literature review that focused on addressing the complexities associated with the selection of PROMs [25].

Then for this specific project, we focused specifically on BC [26]. In this landscape, as well as in several other solid tumors, in recent years the results obtained in clinical research with new drugs have substantially changed the previously applicable therapeutic algorithms, leading to the approval of new treatments in clinical practice. Different PROMs were used in each pivotal trial, so there is a need for a better understanding of the characteristics of the different instruments, both in the field of research and in the recommendations for the use of PROMs in clinical practice.

The primary objective of this study was to develop a comprehensive archive of BC specific PROMs to shed light on their characteristics and specific outcome domains covered.

Furthermore, this project provides a synthesized overview of the current landscape of BC specific PROMs and identifies gaps, strengths, and commonalities.

Ultimately, our goal is to facilitate the selection of more relevant and effective PROMs, thereby ensuring a comprehensive and patient-centered approach to assessing outcomes in BC research, also relying on the use of such PROMs in clinical trials associated with recent regulatory approvals of drugs in patients with BC.

## 2 Material and methods

As part of the PRO4All project, we searched various sources for available PROMs in oncology.

We retrieved all instruments developed by the European Organisation for Research and Treatment of Cancer (EORTC) (qol.eortc.org/questionnaires/). Similarly, we retrieved all measures developed by the Functional Assessment of Chronic Illness Therapy (FACIT) group, with the exception of measures intended for other conditions (facit.org/facit-measures-searchable-library). Additionally, we manually searched the ePROVIDE database (https://eprovide.mapi-trust.org/) and we searched PubMed by using as keywords “patient-reported outcome”, “patient-reported outcome measures”, “caregiver-reported outcome measures”, “cancer”, “oncology”, “review” to retrieve any undetected instruments in the published literature.

Details about methodology have been reported in the paper already published [25].

For this project, we selected only items related to BC, checking in our general database [25] only “*cancer”* in the field “*therapeutic area*” and "*breast cancer*" in the field “*type of cancer”* based on the International Classification of Diseases ICD-10*,* conducting a specific cancer research.

We led searches via Google Scholar, PubMed, and Scopus to identify published articles that reported a validation process or psychometric evaluation or determined the minimum clinically important difference (MCID) [27] for individual instruments, which is an essential step to facilitate the interpretation and use of PROM results for decision making. In fact, the MCID of a PROM represents a threshold value of change in PROM score deemed to represent a clinically relevant improvement.

Finally, the questionnaire items were extracted and assigned to a specific domain according to a predefined taxonomy of 38 outcome classification items, which is used for the outcomes included in all studies, core outcome sets (COS), systematic reviews and trial registries [28]. The assignment of domains was performed by one author (M.M.) and reviewed by other two (F.M. and O.C.); discrepancies were resolved by consensus.

## 3 Results

The initial searches retrieved 132 and 34 questionnaires from FACIT and EORTC websites, respectively. Of these, 7 FACIT instruments were excluded because they were intended for the general population or related to other conditions (FACIT-TB: Functional Assessment of Chronic Illness Therapy – Tuberculosis; FAHI: Functional Assessment of HIV Infection; FAMS: Functional Assessment of Multiple Sclerosis; FACIT-Sp-NI: Functional Assessment of Chronic Illness Therapy - Spiritual Well-Being - Non-Illness; FACIT-SWiP: Functional Assessment of Chronic Illness Therapy - Satisfaction with Pharmacist; FACIT-TS-G: Functional Assessment of Chronic Illness Therapy - Treatment Satisfaction – General; FACIT-TS-PS: Functional Assessment of Chronic Illness Therapy - Treatment Satisfaction - Patient Satisfaction).

**Fig. 1:**
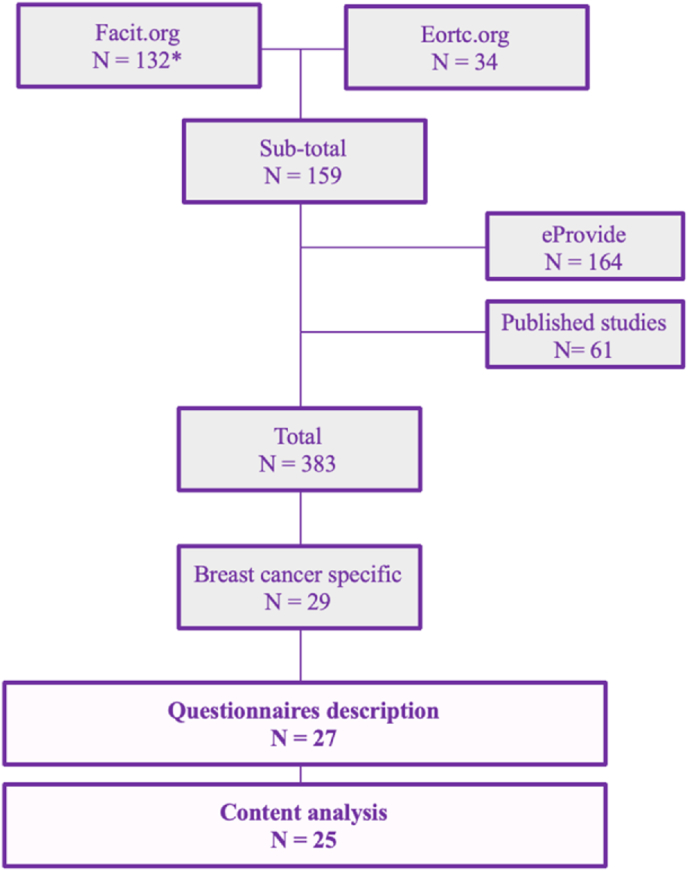
Flow chart of process of breast cancer PROMs selection (∗ 7 measures were excluded from the FACIT group because they were not related to the oncology area*)*.

To these 159 PROMs, 164 additional measures were added from eProvide, 60 from published literature, including reviews and individual studies, reaching a total of 383 PROMs. Of these, 29 were BC specific, but 2 were excluded because the questionnaires description was not available (Satisfaction with Life Domains Scale for Breast Cancer; Subjective Health Estimations). So, we fully examined a total of 27 (7 %) BC specific PROMs (Fig. 1.).

### 3.1 Questionnaires description

All questionnaires (27; 100 %) were self-reported. Besides 21 (78 %) original questionnaires, 6 (22.2 %) were variants (e.g., short versions, parental versions) and there were not duplicates (i.e., same items).

The recall period was “last week” in 6 cases (22.2 %), “today” in 5 (18.6 %), “treatment-related” (e.g. “during your hospital stay", "since your last chemotherapy cycle", "since your last appointment”) in 4 (14.8 %), “last month” in 3 (11.1 %), other in 2 (7.4 %), not specified in 6 (22.2 %) cases and not found in 1 (3.7 %) case.

For 24 instruments (89 %) there was a published study on validation or psychometric properties, but MCID reference was found only in 4 (14.8 %) cases. In particular, Voineskos et al. analyzed BREAST-Q data available of 3052 patients and discovered that the MCID estimates for each domain were 4 (Satisfaction with Breasts), 4 (Psychosocial Well-being), 3 (Physical Well-being), and 4 (Sexual Well-being) [29]. About EORTC QoL questionnaire (QLQ) BR23, Ousmen et al. investigated the impact of the Response Shift (RS) effect on the determination of the MCID over time: in case of deterioration of health related QoL level, an increase of RS effect has been detected in 4/7 dimensions of QLQ-BR23 questionnaire; while, in case of improvement a decrease of the RS effect was observed in 5/7 dimensions of QLQ-BR23 questionnaire. The MCID became ≥5 points when taking into account the RS effect in 5/7 dimensions of QLQ-BR23 questionnaire [30]. Then, Eton et al. showed that MCID estimates were 7–8 points for Functional Assessment of Cancer Therapy-Breast (FACT-B) [31]. Lastly, Li et al. determined MCIDs for Quality of Life Instrument for Cancer Patients - Breast (QLICP-BR V2.0) showing that the MCIDs of the physical domain, psychological domain, social domain, common symptoms and side effect domain, core/general module, specific domain, and the total score were 16.24, 11.37, 11.31, 12.07, 11.49, 10.69, and 11.23 respectively using criteria A (one level improvement after treatment), and 8.88, 15.14, 14.10, 14.50, 13.93, 12.17, and 14.23 respectively using criteria B (at least one level improvement after treatment) [32].

As for the scoring system, 15 (55.6 %) questionnaires had both total and sub scores, while 9 (33.3 %) had only the total score and 3 (11.1 %) had only sub scores.

### 3.2 Content analysis

Out of the total 27 questionnaires, content analysis was conducted on 25 (92.6 %) ones for which the full text was available.

**Fig. 2:**
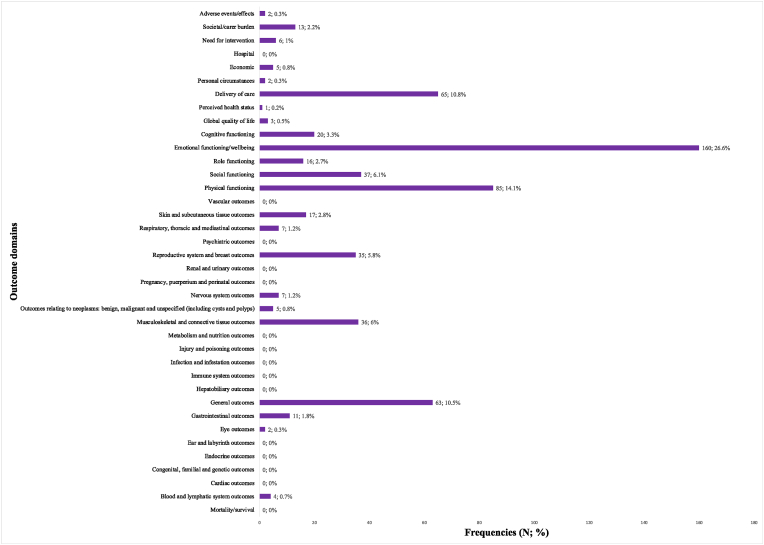
Categorization of the items (n = 602) based on a predefined taxonomy of 38 outcome classification items.

In total, 602 items were assigned to an outcome domain. The most frequent domain was emotional functioning/wellbeing (160; 26.6 % of cases), followed by physical functioning (85; 14.1 %), delivery of care (65; 10.8 %), and general outcomes (63; 10.5 %) (Fig. 2.).

The mean number of items per questionnaire was 25.81 (median 22, range 6–71, interquartile range 12–36).

A list of all questionnaires analyzed is available at link https://cergas.unibocconi.eu/resources/pro4all.

## 4 Discussion

Our in-depth investigation provided an overview of existing BC specific PROMs in oncology: we collected and described 27 PROMs from a search of published reviews, websites and biomedical literature databases. The full text or extracted individual items from associated publications were available for 25 (92.6 %) PROMs, of which 602 items were considered for content analysis using the methodology described previously. This mapping revealed the variety of instruments available in oncology, the existence of variants, different scoring systems and the length of the questionnaires, and showed that the most common outcome domain was emotional functioning/wellbeing one (26.6 % of cases), followed by physical functioning (14.1 %). The same two outcome domains were the two most frequent also in the general database about all cancer types, although the frequency of items related to emotional domain in this analysis specific for breast cancer was even higher compared to the general database (26.6 % vs 21.8 %) [25]. This result reflects the great relevance of emotional aspects and wellbeing in patients with breast cancer, both in the early stages and with advanced disease. The attention to these aspects is important, both in the choice of PROMs and in the presentation and interpretation of QoL results.

In the literature there are some paper about BC specific archives on PROMs, but they refer to a specific subgroups of patient population, unlike our repository referred to the totality of patients with BC, regardless of the disease setting. For example, Ticha et al. have recently published a review mapping PROs in bilateral prophylactic mastectomy with reconstruction, showing that psychological well-being and satisfaction with the breast increased postoperatively [33]. Another study focused on PROMs in breast surgical oncology and reconstruction: it sought to identify the benefits of using PROMs in this setting by comparing 10 instruments and identifying their characteristics [34]. Another review checked 11 PROMs suitable only in metastatic setting of BC [35]. EORTC QLQ-C30 and FACT-B were the most frequently used instruments in these last two studies [34,35].

Our study has thus provided a comprehensive archive of BC specific PROMs that can be very useful both in daily clinical practice and in clinical research. Having a detailed, updated, and useable repository of these instruments is definitely the strength of this research.

At the same time, there are also some limitations.

First, the full text of the questionnaires was not available for all PROMs described, although fortunately this occurred in a minority of cases (7.4 %). Some PROMs are freely available for consultation and for use, some others can be consulted but need permission to be used, while some (like the 2 not available for our analysis) are even difficult to find for consultation because the access is restricted to journal subscribers. This can be a limitation not only for the completeness of our analysis, but also for the availability and use of these instruments.

Second, although we did efforts to explore and search several different sources, we cannot completely rule out the possibility that some PROMs were not identified in our research.

Third, the assignment of results to the 38 areas of taxonomy, made by researchers and a panel of oncologists, may still be subject to subjective judgement.

Fourth, this review cannot help to definitively answer the question of which of the tools described are most appropriate for a particular clinical condition or for a certain research.

The increasing complexity of the PROMs landscape requires a comprehensive overview to enable researchers and clinicians to make an informed decision on the most suitable option on a case-by-case basis for the different needs of patients with cancer. The choice may depend on several variables and considerations.

(a) Some questionnaires may not be validated in the desired language.
(b) It is important to consider patients' compliance in respect of the length of questionnaire: the range of the number of items is indeed very wide (6–71) and questionnaires that are too long could be difficult to use repeatedly. If the completion time ranges from 2 to 3 min, the questionnaire can be more appreciated by patients, and consequently compiled with more dedication and attention. Furthermore, in some cases, investigators use more than one questionnaire in the same study, so the overall burden should be considered in addition to the length of each single instrument.
(c) The fact that MCID reference was found only in 4 (14.8 %) publications could represent a critical issue for clinicians and researchers in the subsequent clinical interpretation of changes in PROs, contributing to the overall reliability of the collected data. In fact, in general, since the clinical relevance of a difference in QoL scores is not immediately perceived and quantifiable, all articles should make explicit, with proper references, the definition of MCID for all the QoL scores analyzed [[29], [30], [31], [32],^36^]. Although the proportion of PROMs with MCID reference is globally disappointing, MCID reference is available for those instruments commonly used in pivotal clinical trials, such as EORTC and FACT questionnaires. Even in this case, however, the availability of a MCID reference does not necessarily imply that both the authors and the readers give adequate relevance to MCID in the interpretation of results.
(d) Although recall period is variable among the PROMs included in the analysis, ranging from the single day of administration to the whole period between the visits, questionnaires commonly used in pivotal trials (EORTC and FACT) are referred to the last week. In specific situations, this should be among the variables considered for the choice of instrument and for the proper interpretation of results.
(e) Another way to help selecting the most appropriate PROM for the scientific or clinical setting is to see which PROMs have been used most frequently in the trials that have led to EMA approval of *practice-changing* drugs in the last 5 years (Table 1.). In some studies, limited to the early disease setting, we have observed the use of PROMs belonging to FACIT group and concerning fatigue, typically a treatment-related adverse event. As a general consideration, being the early disease setting less clinically complex than the metastatic one (in the absence of symptoms related to the disease), proposing questionnaires with items in smaller quantities, but more targeted on specific treatment-related symptoms can be a valid option. In most studies conducted in the metastatic setting, as apparent in Table 1, PROMs belonging to the EORTC group - along with EuroQoL 5 Dimension 5 Level (EQ-5D-5L) - were adopted. The metastatic disease is characterized by women with potentially relevant disease-related symptoms and functional distress. Social and emotional distress can be present in all disease settings, with different determinants, and should be adequately assessed.

**Table 1:** Patient-reported outcome measures used in most relevant practice-changing drugs approved by European Medicine Agency (EMA) for BC treatment in the last 5 years.

Drug approved by EMA	Setting of disease	Trial/ClinicalTrials.gov	PROMs used
Sacituzumab Govitecan	Metastatic BC	ASCENT trial/NCT02574455	EORTC QLQ-C30
Elacestrant	Metastatic BC	EMERALD trial/NCT03778931	EQ-5D-5L
			EORTC QLQ-C30
			PRO-CTCAE
Talazoparib	Metastatic BC	EMBRACA trial/NCT01945775	EORTC QLQ: C30; BR23
Tucatinib	Metastatic BC	HER2CLIMB trial/NCT02614794	EQ-5D-5L
Trastuzumab deruxtecan	Metastatic BC	DESTINY-Breast03 trial/NCT03529110	EORTC QLQ: C30; BR45
			EQ-5D-5L
Alpelisib	Metastatic BC	SOLAR1 trial/NCT02437318	EORTC QLQ-C30
Olaparib	Adjuvant setting	OlympiA trial/NCT02032823	FACIT-Fatigue
			EORTC QLQ-C30
Abemaciclib	Adjuvant setting	monarchE trial/NCT03155997	FACT-Breast 37-item questionnaire
			FACIT-Fatigue
			EQ-5D-5L
Pembrolizumab	Neoadjuvant and adjuvant setting	Keynote 522 trial/NCT03036488	EORTC QLQ: C30, BR23
			EQ-5D

In general, choosing specific BC tools, like EORTC QLQ-BR23 or FACT-Breast 37-item questionnaire with MCID reference, along with the general instruments of the respective “family”, like EORTC QLQ-C30 or FACT-G, could be a good solution in both disease settings.

In conclusion, the main selection criteria of PROM could be summarized in: availability of validation in the specific language; reasonable time for compilation; presence of MCID reference to properly interpret the clinical relevance of results; disease setting (eg early vs advanced).

## 5 Conclusions

Similar to the findings obtained from a general search for PROMs [25], this research highlighted the availability of many instruments specifically developed for BC. The newly developed archive represents a useful tool to optimize the use of PROMs in the evaluation of treatments in patients with cancer, promoting a patient-centered approach both in clinical research and practice.

## Funding

This work was supported by a research grant (PRO4All) from 10.13039/100014805Roche Italy to CERGAS (10.13039/501100005300SDA Bocconi).

## Ethical approval

Not required.

## CRediT authorship contribution statement

**Anna Amela Valsecchi:** Writing – original draft, Validation, Methodology, Investigation, Formal analysis, Data curation, Conceptualization. **Filippo Giovanardi:** Writing – original draft, Validation, Methodology, Investigation, Formal analysis, Data curation, Conceptualization. **Francesco Malandrini:** Writing – review & editing, Validation, Supervision, Project administration, Methodology, Investigation, Funding acquisition, Formal analysis, Data curation, Conceptualization. **Michela Meregaglia:** Writing – review & editing, Validation, Supervision, Project administration, Methodology, Investigation, Funding acquisition, Formal analysis, Data curation, Conceptualization. **Alberto Servetto:** Writing – review & editing, Validation, Data curation, Conceptualization. **Chiara Bennati:** Writing – review & editing, Validation, Data curation, Conceptualization. **Carmine Pinto:** Writing – review & editing, Validation, Data curation, Conceptualization. **Massimo Di Maio:** Writing – review & editing, Validation, Supervision, Project administration, Methodology, Investigation, Funding acquisition, Formal analysis, Data curation, Conceptualization. **Oriana Ciani:** Writing – review & editing, Validation, Supervision, Project administration, Methodology, Investigation, Funding acquisition, Formal analysis, Data curation, Conceptualization.

## Declaration of competing interest

**F.G.** reports honoraria from Eli Lilly, MSD, AstraZeneca, Novartis, Pfizer, Roche, Tesaro/GlaxoSmithKline.

**A.S.** reports honoraria from Eli Lilly, MSD, AstraZeneca and Janssen; Travel support from Bristol-Myers Squibb, Roche and AstraZeneca; funding for research from AIRC.

**C.P.** reports honoraria from Amgen, Astellas, AstraZeneca, Bayer, Bristol Meyer Squibb, Celgene, Clovis Oncology, Eisai, Ipsen, Janssen, Incyte, Merck-Serono, Merck Sharp and Dohme, Novartis, Roche, Sandoz, Sanofi, and Servier for consultancy or participation to advisory boards.

**M.D.M.** reports honoraria from 10.13039/100002429Amgen, 10.13039/100004325AstraZeneca, Boehringer Ingelheim, 10.13039/100004330GlaxoSmithKline, Janssen, 10.13039/100004334Merck Serono, 10.13039/100009947Merck Sharp & Dohme (10.13039/100030732MSD), 10.13039/100004336Novartis, 10.13039/100004319Pfizer, 10.13039/100004337Roche, Takeda for consultancy or participation to advisory boards; direct research funding from Tesaro/10.13039/100004330GlaxoSmithKline, institutional funding for work in clinical trials/contracted research from Beigene, 10.13039/100010544Exelixis, 10.13039/100030732MSD, 10.13039/100004319Pfizer and 10.13039/100004337Roche.

**A.A.V.**; **C.B.**; **F.M.**; **M.M.**; **O.C.** have declared no conflicts of interest.
